# Human infection with Seoul orthohantavirus in Korea, 2019

**DOI:** 10.1371/journal.pntd.0009168

**Published:** 2021-02-22

**Authors:** Changmin Kang, Jin Il Kim, Jungmin Lee, Seongman Bae, Min Jae Kim, Ki-Joon Song, Jin-Won Song, Sung-Han Kim, Man-Seong Park

**Affiliations:** 1 Department of Microbiology, the Institute for Viral Diseases, Korea University College of Medicine, Seoul, Republic of Korea; 2 Biosafety Center, Korea University College of Medicine, Seoul, Republic of Korea; 3 Department of Infectious Diseases, Asan Medical Center, University of Ulsan College of Medicine, Seoul, Republic of Korea; SCI Foundation, UNITED KINGDOM

## Abstract

Of various rodent-borne hantaviruses, Seoul orthohantavirus (SEOV) causes haemorrhagic fever with renal syndrome (HFRS), as does Hantaan orthohantavirus (HTNV). Given global-scale of cases of human infection with SEOV, it is of great clinical importance to distinguish SEOV from other HFRS-causing hantaviruses. In May 2019, a middle-aged patient who had lived in a suburban area of Chungcheong Province, Republic of Korea and enjoyed outdoor activities was transferred to Asan Medical Center in Seoul, Republic of Korea with HFRS; his symptoms included high fever and generalized myalgia. The rapid diagnostic test performed immediately after his transfer detected HTNV-specific antibodies, and the patient was treated accordingly. However, two consecutive IFAs performed at ten-day intervals showed no HTNV-specific immunoglobulin (Ig) G. During continuous supportive care, next-generation sequencing successfully identified viral genomic sequences in the patient’s serum, which were SEOV and not HTNV. Phylogenetic analysis grouped the L, M, and S genes of this SEOV strain together with those of rat- or human-isolated Korean strains reported previously. Given global outbreaks and public health threats of zoonotic hantaviruses, a causative pathogen of hantavirus HFRS should be identified correctly at the time of diagnosis and by point-of-care testing.

## Author summary

Rodent-borne Seoul orthohantavirus (SEOV) has provoked human cases from Asia to the Americas and Europe whereas most orthohantaviruses cause regional cases. Despite this, SEOV gets less attention than other orthohantaviruses. In Korea, 2019, a middle-aged man was initially diagnosed with Hantaan orthohantavirus (HTNV) and treated accordingly. However, next-generation sequencing identified SEOV, not HTNV, in the patient’s serum. Given its global outbreaks and public health threats, zoonotic SEOV should be diagnosed correctly on point of care to reduce unnecessary medical costs.

## Introduction

Hantaan orthohantavirus (HTNV) of the family *Hantaviridae* was first isolated as the cause of Korean haemorrhagic fever in 1976 [1]; since then, 36 species within the genus *Orthohantavirus* have been identified from various rodent species in many different regions [2,3]. Some of these viruses cause haemorrhagic fever with renal syndrome (HFRS) in humans; others may cause severe respiratory disease, such as hantavirus pulmonary syndrome (HPS) [4].

Among HFRS- and HPS-causing orthohantaviruses, Seoul orthohantavirus (SEOV) is the only hantavirus that has caused many human cases (more than 50 cases detected by an immunofluorescence assay or enzyme-linked immunosorbent assay until 2019) in a global scale, from Asia to Africa, the Americas, Europe, and Oceania (Table 1) [5]. In Europe, particularly in Belgium, France, the Netherlands, the United Kingdom, and Sweden, SEOV human infection has been associated with the brown rat, *Rattus norvegicus*, a natural reservoir of SEOV [5]. Moreover, it has been reported that in addition to wild rats, both pet and laboratory rats can transmit SEOV to humans in the United States and Canada [6]. Travellers can also contract SEOV in endemic areas [7] and spread the virus. Even though SEOV often results in mild illness in humans, the increasing number of human cases of SEOV infection has been reported recently [8–10]. However, transmission dynamics of zoonotic SEOV still remain elusive [11,12]. Given these epidemiological and public health concerns, SEOV may pose a more severe threat than other orthohantaviruses.

**Table 1:** The number of SEOV cases in rats and humans reported until 2019.

	^a^The number of SEOV cases in rats/humans^b^ based on the detection method^c^			
Region	IFA or ELISA	Neutralization assay	RT-PCR	Virus isolation
Asia (including Oceania)	22/23	9/8	17/10	18/4
Europe	12/11	5/0	11/7	4/0
Americas	2/5	5/7	3/4	4/0
Africa	6/15	1/0	0/0	1/0
Total	42/54	20/15	31/21	27/4

^a^ modified from the figures presented in Clement et al.’ review article [5].

^b^ the number of rat cases/human cases.

^c^ IFA, immunofluorescence assay; ELISA, enzyme-linked immunosorbent assay; and RT-PCR, reverse transcription-polymerase chain reaction.

SEOV was first isolated from an urban brown rat in Seoul, Republic of Korea, in 1980 [13]. However, it is difficult to discriminate SEOV from HTNV because both viruses cause similar clinical symptoms in humans, with close relatedness in serological assays [5]. This suggests the possibility of HFRS misdiagnosis, and HTNV-like viruses, but not SEOV, have been considered the agents responsible for most cases [5,14]. An epidemiological history might also affect the diagnostic approaches used for HFRS patients [15], as occurred in our case. In this regard, we here emphasized that SEOV, as well as HTNV-like viruses, should be prioritized as one of the pathogenic agent of HFRS and investigated molecular evolution patterns of rodent and human SEOV strains including the complete genomic sequences of SEOV isolated from the HFRS patient specimen using a next-generation sequencing (NGS) method.

## Methods

### Ethics statement

This study was approved by the Institutional Review Board in Asan Medical Center, University of Ulsan College of Medicine (approval number: 20160748), and written informed consent was obtained from the patient.

### Diagnosis

Using clinical specimens (whole blood and serum) collected from the patient during hospitalization in Asan Medical Center, a rapid diagnostic test (ImmuneMed Hanta Rapid, Immune Med Inc., Chuncheon, Republic of Korea) was performed according to the manufacturer’s protocol. The ImmuneMed Hanta Rapid test is a lateral flow immunochromatographic assay that uses the hantavirus nucleocapsid recombinant protein as the antigen. The test was performed by inoculating 3 μl of serum or 6 μl of whole blood into the kit and then adding 7 drops of sample buffer (300 μl). The results were visualized after 15 minutes by a clinician.

### Immunofluorescence assay

Serum antibodies against HTNV, including immunoglobulin (Ig) G and IgM, were measured by indirect IFA (Green Cross Reference Laboratory, Yongin, Republic of Korea). A titer of ≥40 was considered positive. In brief, pre-manufactured IFA slides stored at -80°C were thawed at room temperature for 30 minutes before the experiment. Samples were serially diluted to a 1:40 concentration with phosphate-buffered saline, added to each well of the antigen slide, and incubated at 37°C for 30 minutes. Then, 20 μl of FITC-conjugated anti-human IgM and IgG was added to each well and allowed to react at 37°C for 30 minutes. The results were observed under a fluorescence microscope at 400× magnification.

### Next-generation sequencing

Viral RNA was obtained from patient serum samples (QIAamp viral RNA mini kit: QIAGEN, Hilden, Germany) and used for sequence library construction using an Illumina TruSeq RNA sample prep kit v2 (Illumina, San Diego, CA, USA). Before sequencing, the quality and quantity of the genomic library were evaluated using the KAPA Library Quant Kit (Illumina). The quantified library was sequenced by an Illumina MiSeq 150-bp paired-end platform (Illumina), and the sequencing output data were analyzed using CLC Genomics Workbench 10 (QIAGEN). After removing low-quality reads, a total of 7,937,243 reads out of 8,137,452 reads (approximately 97.5%) were mapped to the consensus rat-origin SEOV genome sequences downloaded from GenBank.

### Phylogenetic analysis

The National Center for Biotechnology Information (U.S. National Library of Medicine, Bethesda, MD, USA)-registered full-length nucleotide sequences (for the L gene, n = 21; M, n = 31; and S, n = 34) of SEOVs were used as references. Each sequence set was aligned using MAFFT [16], and phylogenetic relationships were reconstructed using MEGA X [17] based on the initial trees obtained by the neighbor-joining method through the maximum composite likelihood approach. For the nucleotide substitution models, GTR + G + I was selected for all three gene segments, and the bootstrap scores were indicated with different colors at the tip of each node, with 1,000 replicate estimations. The maximum likelihood (ML) trees were then visualized using FigTree (v1.4.4; accessed through https://groups.google.com/forum/#!forum/figtree-announce).

## Results

**Fig 1:**
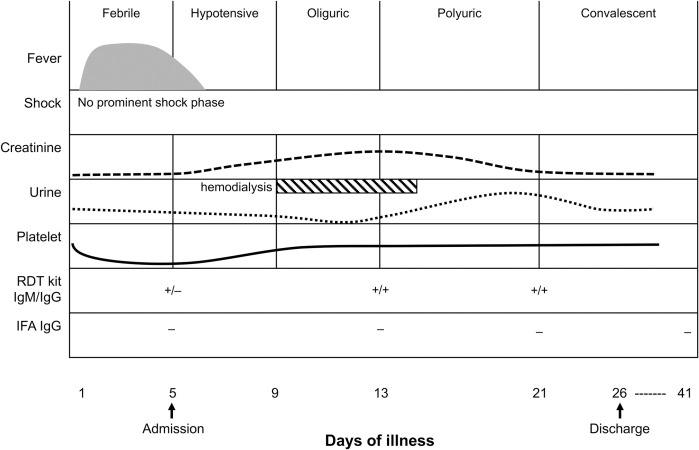
Disease courses and profiles of clinical and diagnostic examinations. Given the disease courses (febrile, hypotensive, oliguric, polyuric, and convalescent), clinical examinations (fever, shock, creatinine, urine output, and platelet levels), treatment (haemodialysis) and diagnostic tests (rapid diagnostic test and immunofluorescence assay) were performed on the indicated days of illness.

In May 2019, a 55-year-old man who lived in a suburban area of Chungcheong Province, Republic of Korea, so that he could enjoy outdoor activities was transferred to Asan Medical Center in Seoul with high fever (39°C) and generalized myalgia. He had been farming in the field in front of his house every weekend. He did the field work on weekend, as usual, 7 days before symptoms appeared. On May 19, he suddenly developed fever, chills, and generalized myalgia and was admitted to the hospital near where he lived. During the hospitalization, fever persisted and bicytopenia (leukopenia and thrombocytopenia) developed. On May 23, he was transferred to Asan Medical Center, a tertiary referral hospital, to seek advanced diagnostic tests and opinion of infectious diseases expert. The clinical data presented in Fig 1 were retrieved from the patient’s medical record.

After admission (on day 5 of his illness) in Asan Medical Center, the patient exhibited HFRS-like symptoms, such as leukopenia, thrombocytopenia, elevated hepatic enzymes, fever (38.8°C), chills, headache, and generalized myalgia. Laboratory findings were as follows: white blood cells, 3.3×10^9^/L; platelets, 32×10^9^/L; aspartate aminotransferase, 68 U/L; alanine aminotransferase, 55 U/L; creatinine 0.90 mg/dL; and C-reactive protein 2.5 mg/dL. A rapid diagnostic test detected HTNV-specific immunoglobulin (Ig) M and IgG antibodies. However, no HTNV-specific IgG was detected by IFA (Fig 1). Given the clinical manifestations and history of outdoor activities, empirical doxycycline and ceftriaxone were administered on day 8 of his illness to treat potential scrub typhus and leptospirosis. The patient also experienced oliguric renal insufficiency, and on day 10, the renal involvement worsened, as his urine output decreased to 200 cc/day. Despite continuous supportive care, the urine output did not exceed 500 cc/day until day 15 of his illness; another 10 days elapsed before the urine output recovered to a normal level. On day 21, the rapid test still showed positivity for HTNV IgM and IgG, whereas IFA detected no HTNV-specific antibodies. The patient was discharged on day 26 and visited an outpatient clinic two weeks later, but no renal complications were observed (Fig 1).

**Fig 2:**
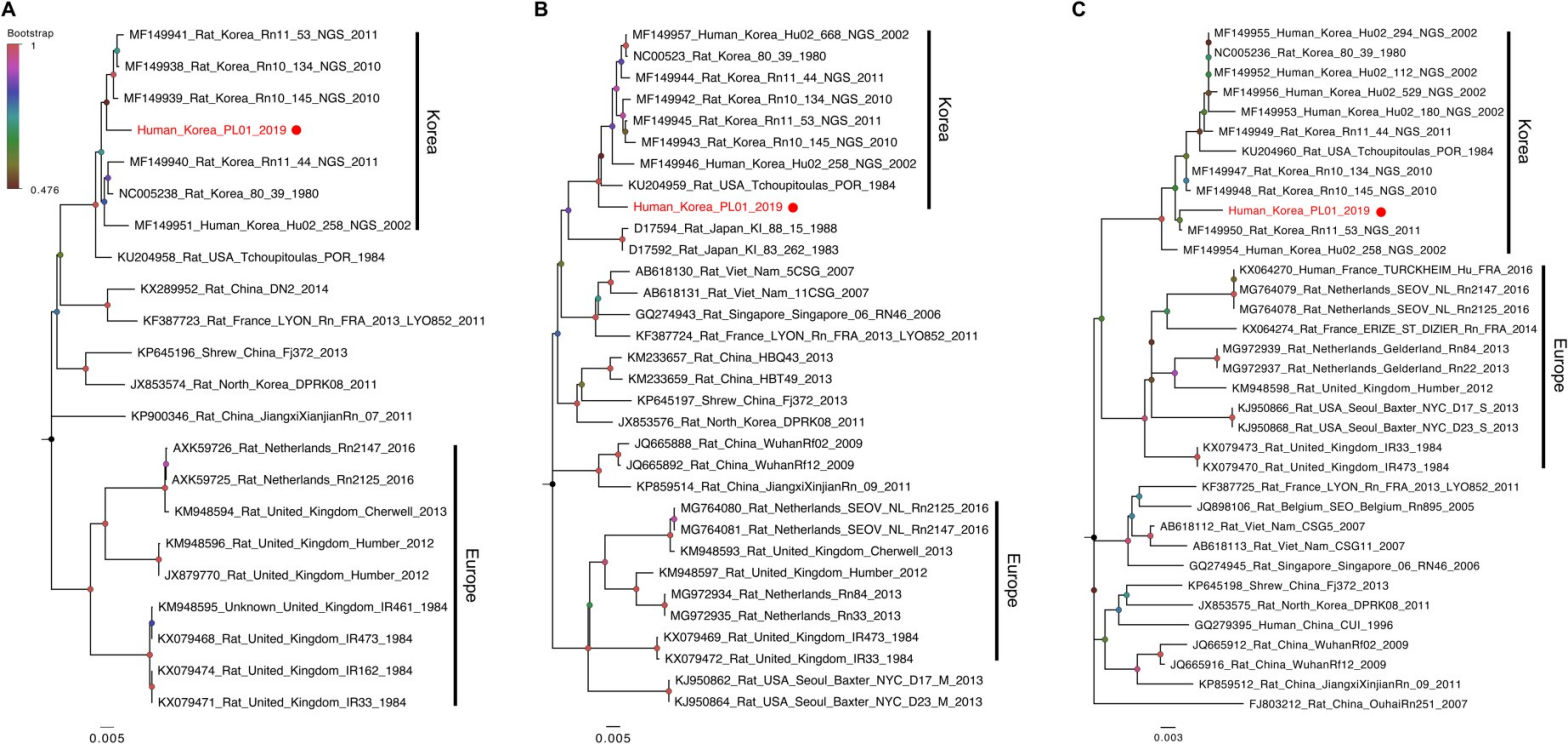
Phylogenetic relationships of the L, M, and S gene segments of SEOVs. Phylogenetic relationships of the L (A), M (B), and S (C) gene segments of SEOVs were reconstructed based on reference sequences downloaded from the NCBI database, and the ML trees indicate two large groups from Korea and Europe. Each gene sequence of PL01 SEOV is coloured red and additionally indicated with a red circle. Bootstrap scores (0.48 to 1) are indicated with different colours at the tip of each node. The scale bar indicates the mean number of nucleotide substitutions per site.

Although the patient fully recovered from HFRS, we were not able to identify a causative pathogen based on routine tests. Instead of serological assays, we used an NGS method, and intriguingly, a *de novo* genome assembly algorithm identified genetic sequences of SEOV, not those of HTNV, in the patient’s serum. Using designed extension primers, we obtained the full-genome sequences of the L, M, and S genes of SEOV (Human_Korea_PL01_2019; PL01) (S1 Data). Furthermore, phylogenetic analysis grouped the L, M, and S genes of PL01 together with those of rat- or human-isolated Korean SEOVs, including the closely related strain Rat_USA_Tchoupitoulas_PRO_1984 (Fig 2) [18,19]. Unlike the clustering patterns of the L and M genes, the S gene sequences of PL01 and other Korean strains appear to be more closely related to those of European strains than to those of Chinese and southeastern Asian strains (Fig 2), indicating potential reassortment events between the L, M, and S gene segments of rodent and human SEOV strains.

## Discussion

Given its natural hosts and global-scale cases of human infection [5], SEOV should be considered a public health threat with potential for global outbreaks. Although SEOV was first reported in Asia, it has now reached the Americas, Europe, and even Oceania (Table 1) [5–7,13,20], and the virus can infect wild, pet, and laboratory rats, and may then transmit to humans [6]. Even, travellers may contract SEOV in certain endemic areas [7] and travel around to their destinations. Considering the presence of various HFRS- and HPS-causing hantaviruses in different geographical regions [3] and the unknown transmission dynamics of SEOV, adding SEOV to the top of endemic pathogen lists may be warranted, highlighting the importance of differential diagnosis of SEOV from other hantaviruses.

In our case, the patient was initially considered to be infected with HTNV based on his clinical symptoms and the results of serological assays. Nonetheless, the exact pathogenic cause was not confirmed by repeated diagnostic tests (Fig 1), and preventive antibiotics were prescribed. If we had not considered an NGS approach, this case might be categorized as of unknown cause with acute kidney injury and renal insufficiency, and it is why we emphasize that SEOV should be prioritized as one of the pathogenic agents causing HFRS and similar renal diseases in this study.

The NGS approach allowed us to determine the cause of the HFRS symptoms in our patient. It also provided us with the molecular epidemiologic history of the complete SEOV gene segments [15,18]. Indeed, based on the evolutionary relationships reconstructed (Fig 2), we were able to track potential genetic reassortment events between different rodent and/or human SEOV strains. However, if more cases have been investigated, we might provide a more comprehensive picture of the molecular evolution of SEOV as well as its transmission dynamics between natural reservoir rats and humans. Despite this limitation, our results suggest that a correct diagnostic method that distinguishes SEOV from other hantaviruses in humans and natural hosts will be an important breakthrough in curbing potential SEOV outbreaks and reducing unnecessary medical costs.

### Learning points

- This study reports an important clinical case that SEOV, as well as HTNV, is one of the main causes of HFRS.
- Given the serological cross-reactiveness between SEOV and HTNV, next-generation sequencing can be a diagnostic option for the identification of a HFRS causative agent.
- Phylogenetic analysis using the complete genomic information of SEOV may also provide a knowledge of the evolution and transmission dynamics between rat and human SEOV.

## Supporting information

S1 Data Genetic sequences of PL01 SEOV. Complete sequences of the L, M, and S gene segments of PL01 are provided in the FASTA format.
(DOCX)
