# Supplementary material for: Human infection with Seoul orthohantavirus in Korea, 2019
Source: PLoS Negl Trop Dis. 2021 Feb 22;15(2):e0009168. doi: 10.1371/journal.pntd.0009168 (PMC7932522; doi:10.1371/journal.pntd.0009168)
Supplement: S1 Data — Complete sequences of the L, M, and S gene segments of PL01 are provided in the FASTA format. (DOCX) [file pntd.0009168.s001.docx]

**S1 Data. Genetic sequences of PL01 SEOV.**

>SEOV_Human_Korea_PL01_2019_Lgene

gtggaatgcatggattacttggataggctctatgctgtccggcatgacattgttgaccagatgataaaacatgaatggtcagacaataaagacagtgaggagccaataagtaaggtattgctgtttgctggggtacctaataatgtcataacagcattggaaaaaaaggttatccctgatcatccaagtggtaaaacactgcgatctttttttaagatgacacctgacaactatcgaataacaggttccttaattgagtttgtggaggtaactgtgactgcagatgtagataagggtatcagagagaagaaaatgaaatatgagctaggtctgaaatatttagagcaagaactcatgacattttttcataggggagaactacaaaacccttataaaattacatttaaagtagttgcagtccggacagatggatcaaatatttcaacacaatggcccagtacaaggaatgatggtgtagttcaatacatgagattggtacaggcagaaatcagttatgtaagggaacatctggttaaaactgaagaaagagctgcattagaagcaatgtttaacttgaaattcaacataagttctctaaagacacagccttattttatccctgaatataaagggattgatctgattagacctgatatagatgggttagtgaactatgcccaaagctggatgtcaaagacccaagaattctctttctttgaagtaaaaggttcagctgtgtttgattgttttaatgagaatgaacaagggcacattgtaaaatacccaatgtcccggcacccgagaaactttttactcatccaatgcactgtattaacagcatacaaacctgcaactatactttctgatcaacttgatagccgtagggcctgcattcaatttttaaacctcatcccagaaacccctgcatctattcttgcacatgatatggcacacagatacatcaacttgactagagatgatttattagcttattatgcacctagaatccaatttaacccaacccaaaatattaaagaaccaggcacctttaaattaacatcaaatatgatgaggcctgagtcaaagatcatgctagatatgctaagtcaacatgaacctcgagaaaatctggggaagtcaatagaaagtttaaacatcagtagtcatattgtacagtcagattgtgttagcttaatcacaaaaattttgtcagatttagagcttaatatttctgaacctagtagccatgagcagattacagctaaacacacacatgttgacactgtactggataaattttttcaaaacgagactcagaagtatttaattgatattctaaagaaaaccacagcatggcatatcgggcatttagttagggatattactgagagcttaattgcacattctggattgaggaggtctaaatattggtcaattcatgcttataataatggaagtgtaatattgttcatactcccttcaaaatcccttgaggttgcaggttcgtttgtgcgatttatgactgcttttaaattaggtcctggactagttgataaagacaatttggattcaatacttgcagatggtgatatcttatggggtgtctcaaagattatgagtctagatctgaacaggttacttgctttgaatatagcttttgagaaagcactacttgcaacagctacatggttccaatattacactgaggatcagtcacaatttccattgcaacattctattcggtcagtatttgcttatcattttttgcttgctatttgccaaaagatgaaattatgtgcaatctttgacaacctaaggtatcttatacctgctgtgacatctttgtattctggttttccttctttggttgaaaaactgttcgagagaccttttaaaagtgctttagaagtctatgtgtattataacatcaaaagtctgcttgtcgcactggctcaaaataataaggctagattttattcaaaagttaaactcctcggattgactgtcgaccaatctacagttggtgcaagcggtatctatccatcctttatgtcacgagttgtttataaacattacaagagtttaatttcagaagtgacaacttgcttttttctttttgaaaagggtttgcatgggaatgtgaatgaagaggctaaaatacatctggagactgtagaatgggcaacaaagttcaaagaaaaggaggataagtatggtgagatgttagttgaacatggttatacaataggagagcttgtggaatccagtgaactagcagtccaacagttatattgtcaagatgcagttgagctggctgccaatgaattgaaccgagttcttatcacaaagtctcaggtggttgccaatagtatcttaaataagtattgggaagagccatattttagccaaacaaggaatattagtttaaaaggcatgtcaggccaagtacaagaggatgggcatttgtcgtcatccactacaataattgaagctataagatatctgtctaattcaaggaacaatcctaacgttcttcaactatatgaagaaactagacatcagaaggcacaagctagaattgtgaggaagtttcagaggacagaagctgatcgaggttttttcattacgacactaccaacaagatgtcgtcttgagattattgaggactattatgatgcaatatcaaaaaacgtagcagaggaatatatctcatatggtggagaaagaaaaattctctgtatccaggcagcccttgaaaaagctctccgctgggcatctggagagagctttattgagctcagtaacgggaaatttataaggatgaagagaaaactgatgtatgtcagtgctgatgccactaaatggtctccaggtgataattcagcaaaattcaggcgattcactgctgcccttcataatggactgcctgatgacaggttgaaaaactgtgtcattgatgccttgcgccatgtatataagactgatttttatatgtctagaaaactcagacactatattgattctatggatacttatgaacctcatgtcagagactttttgaatttttttccggacgggcatcatggagaggtacgaggcaattggttgcagggtaacttgaacaagtgttcgtcattgtttggtgtggcaatgtccttattatttaaagaaatttggacaaggttatttccagaattagactgcttttttgaatttgctcatcattcagatgatgcacttttcatatatggctacttagaaccagctgatgacgggactgattggttcttatttgtatcccagcagattcaagctggaaaattgcattggtttaatgtaaatacagagatgtggaaaagtatgtttaatctgcatgaacacattcttctgttaggttcaattaaaatatccccaaagaagacaacgttatcgccgacaaatgctgagtttttgtctacattttttgagggttgtgctgtatcaattcccttcattaaaatccttcttggctcactatctgacctaccaggtcttggttatttcgatgatcttgctgctgcccagacgaggtgcgtcaaagcaatggatttaggagcatcacctcaaatctcacaacttgcagtctcacttagtacgagtaaggttgagagattatatgggacatcaataggcatggttaattatcctggtacatatttaaggacaaaacactctgaaactccaattccactgggaggaagtggtgcaatgtcaataatggaactttcaacagctggtataggtatgtctgacaaaaatttattgaaacaagcacttattggttatatgcataaacaccaaaaacaaatgagttacatcttggggctcttcaagtttttaatggatctctctggtgagacatttcagcatgaaaggctaggccagttctcatttataggaaaagttcaatggaagatattcacacccaaatcagagtttgaattctctgacatgtattctcagaagtttttaaaggtttggagtgaacagcatcctacatatgattacattattcctagaggtcgtgataatcttcttatttatcttgtgagaaaattgaatgacccaagcattataacagctatgactatgcaatcacccttacaactcagatttaggatgcaagcaaaacaacatatgaaggtttgcagactagatggggactgggtaacatttagagaggtacttgcagcagcaaacagcttcgctgagagctatgaacctagtcaaaatgatattgatttatttcagacattgactagctgcacattttctaaagagtatgcatggaaagattttttgaataatgtacactgtgatgtgataccaacaaagcaagtacaacgtgcaaaagttgcacgtacattcacagtgagagaaaaggaccaaattattcaaaacagcattccagctgttattggttacaagtttgcagtaacggttgatgaaatgtctgatgtgctagacacagcaaagttcccagattctctggcagttgacctaaagacaatgaaagatggagtctatagagaattgggtttagatatctcttccccagatgttatgaagaaagtggcacctatgttgtataaaagtgcgaaatcgcgggttgtcattgtccaggggaatgtggaaggaactgcagaggcaatctgtgcatactggttgaggaatatgtcattgattaagacaattaaagttaaaccccataaagaagtgctccaggctgtatccatatttaataggaaagaagatatcggtcaacagaaggatttgtcagcactaaaattatgtattgaagtttggaggtgggcaaaagcaaataatgcaccatatagggactggttccatgcattatggtttgaagacaaaacattttcagagtggctagatagatttatcagagtaggtgtgcctcctattgatccagaaattcagtgtgcagcattgatgatcgcagatgtcaaaggtgacaggtcagtgttacaactccaagccaatagaagagcatattcagggaaacaatatgatgcatattgtgtacagacttataatgaagagacaaagctttatgaaggggatctaagagtaaccttcaattttggattagattgtgcacggcttgagatattttgggataaaaaaacttatattcttgagacatccatcacacagaaacatgttttaaaaatcatgatggaggaggtttcgaaagagctagttaggtgtgggatgcgatttaatactgagcaagttaatggtgtaaagcaccttgttttgttcaagacagattcaggttttgaatggggtaagcctaatattccctgtattgtatacaaaaactgtgcattgaggacaggtttgaggacaaaccaagctatcaaccataagtttatgattacaattaaggatgatggtctcagggcaattgcacagtatgatgaagatagcccacggtttcttttagcacatgctttccatactatccgtgatgttcgctatcaagcagttgatgcagtaagtaatgtgtggttcacacataaggggataaagctatacttgaacccgatcatttcatcaggactgctggagaactttatgaaaaatatacctgctgctattccaccagctgcttattcattaataatgaatcgtgcaaaaatttctgtggacctttttatgtttaatgacttacttagactaataaacccaggtaacacacttgacttgtctggtcttgaactaacaggtgaaggttatagtacggtgagtagtctttctagtagattatggtcagaagaaatgagccttgttgatgatgaagaggagatggatgatgagttcacaatagacctacaggatgtagattttgaaaatattgatatagaagctgatgtagagcatttcttacaggatgagagtgcttatacaggtgatttattgattatgtcagaagaaacagaagtaaaaaagatgagagggataattaagcttttagaacctgtaaagttaataaagagctgggtttcaaggggactgtctattgaaaaggtatacaacccagttaacatcatactaatgaccaggtatatatcaaaaaactttaacttttcaggcaagcaggtatccttattagacccatatgatttaactgagcttgagagtattgttaagggatggggtgagtctgttgtggatcagtttgattcccttgacttagaagcccagaatctagttcaaaagcagggtattgtacctgaggatgtgattccagattctttgttttcctttagacatactatggtactattacgccggctgtttccacaggattcagtttctacattctat

>SEOV_Human_Korea_PL01_2019_Mgene

atgtggagtttgctattactggccgctttagttggccaaggctttgcattgaaaaatgtgtttgacatgagaattcagtgtccccattcagtcaactttggggaaacaagtgtgtcaggctacacagaactgcccccactttcattacaggaggcagaacagctagtgccagagagctcatgcaacatggacaaccaccaatcactctcaacaataaataaattaaccaaggtcatatggcggaaaaaggcaaatcaggaatcagcaaaccagaattcatttgaagttgtggagagtgaagtcagctttaaaggattatgtatgttaaagcatagaatggttgaagaatcatacagaaataggagatcagtaatctgttatgatctagcctgtaatagtacattctgtaagccaactgtctacatgattgttcctatacatgcatgcaacatgatgaaaagctgtttgattggtcttggtccctacagaatccaggttgtttatgaaaggacatactgcactacgggtatattgacagaagggaaatgctttgttcctgacaaggctgttgtcagtgcattgaagagaggcatgtatgccatagcaagcatagagacaatctgcttttttattcatcagaaagggaatacatataagatagtgactgccatcacatcggcaatgggctccaaatgtaataatacagatactaaagttcaaggatattatatctgtattattggtggaaactctgcccctgtatatgcccctgctggtgaagactttagggcaatggaggttttttccgggattattacatcaccacatggagaagaccatgacctccctggcgaggaaattgcaacataccagatttcagggcagatagaggcaaaaatccctcatacagtgagctccaagaacttaaaattgactgcctttgcaggtattccatcatactcatcaactagtatattggctgcttcagaagatggtcgtttcatatttagtcctggtctatttcctaacttaaatcagtcagtctgtgacaacaatgcactccctttaatctggaggggcctaattgatttaacgggatactatgaagcagttcacccttgcaatgtattctgtgtcttatcaggaccaggtgcttcatgtgaagccttttcagaaggaggtattttcaatattacttccccaatgtgtcttgtgtccaagcaaaataggtttagagcagctgagcagcagattagctttgtctgccaaagggttgatatggatattatagtgtactgtaatggtcagaaaaagacaatcctaacaaaaacattagttataggccaatgcatttatactattacaagtctcttttcactgttaccaggggttgcccattctattgctattgagttgtgtgttccaggatttcatggctgggccacagctgcacttttgatcacattctgctttggctgggtattgattcctgcatgtacattagctattcttttagttcttaagttttttgcaaatatcctccatacaagcaatcaagagaaccgatttaaagccattctacagaaaataaaggaggagtttgaaaaaacaaagggttccatggtttgtgagatctgtaagtacgagtgtgaaacattaaaggaattgaaggcacataatctatcatgtgttcaaggggaatgcccatattgctttacccactgtgaaccgacagaaactgcaattcaagcacattacaaagtttgtcaagctacccaccgattcagagaagatttaaaaaagactgtaactcctcaaaatattgggcctggttgttaccgaacattaaatctttttaggtataaaagtaggtgttatattctgacaatgtggactcttcttctcattattgaatccatcctctgggcagcaagtgcagtagaaatcccccttgtccctctctggacagataatgctcatggtgttgggagtgttcctatgcatacagatcttgaattagacttttctttaccatctagttctaagtacacatataaaagacatcttacaaacccagttaatgaccaacagagtgtctcattgcatatagaaattgaaagtcaaggcattggtgctgatgtccatcatcttggacattggtatgatgcaagattgaatttaaaaacctcatttcattgttatggtgcctgcacaaaatatcaatatccatggcacactgcaaaatgccattttgagaaagattatgagtatgaaaacagctgggcatgcaaccccccagattgcccaggggttggtacaggctgtactgcttgtgggttatatcttgatcaattgaagccggtagggacagccttcaaaatcataagtgtaagatacagtagaaaagtgtgcgtgcagtttggtgaagaatacctttgtaaaacaattgatatgaatgattgctttgtgactaggcatgccaaaatatgtattattgggactgtatctaagttttctcaaggtgacactctactatttctagggcccatggaaggaggtggtataatctttaaacactggtgcacatcaacctgtcactttggagaccctggtgatgtcatgggtccaaaagataaaccatttatttgccctgaattcccagggcaattcaggaaaaaatgtaactttgccacaactccagtttgtgaatatgatgggaacattatctcaggctataagaaagttcttgcaacaattgattctttccaatcatttaacacaagcaatatacacttcactgatgagagaattgaatggagagaccctgatggtatgcttcgggatcatattaatatcgttatttctaaagatattgattttgaaaatttggctgagaatccttgtaaagtagggctccaagcagcaaacatagaaggtgcctggggttcaggtgtcgggtttacactcacatgccaggtgtccctcacagaatgtccaacatttctcacgtcaataaaggcctgtgacatggcaatttgttatggtgcagaaagtgtgacactctcacgaggacaaaatactgtcaaaattactgggaaaggtggccatagtggttcctcatttaagtgctgtcatgggaaagaatgttcattaactggactccaagccagtgcaccacatttagataaagtaaatggtatctctgagttagaaaatgagaaagtttatgatgatggtgcacctgaatgtggcattacttgttggtttaaaaaatcaggtgaatgggttatgggtataatcaatgggaactgggttgtcctaattgtcttgtgtgtcctgctgctcttttctcttatcctgttgagcatcctgtgtcctgttaga

>SEOV_Human_Korea_PL01_2019_Sgene

gcagcttcaatacaatcaaaaattgatgaactgaagcgccaacttgccgacaggattgcagcagggaagaacatcgggcaagaccgggatcctacaggggtagagccgggtgatcatctcaaggaaagatcagcactaagctacgggaatacactggacctgaatagtcttgacattgatgaacctacaggacagacagctgattggctgaccataattgtctatctaacatcattcgtggtcccgatcatcttgaaggcactgtacatgttaacaacaagaggtaggcagacttcaaaggacaacaaggggatgaggatcagattcaaggatgacagctcatatgaggatgtcaacgggatcagaaagcctaaacatctgtatgtgtcaatgccaaacgcccaatccagcatgaaggctgaagagataacaccaggcagattccgcactgcagtatgtgggctatatccggcacagataaaggcaaggaatatggtaagccctgtcatgagtgtagttgggtttttggcactggcaaaagactggacatctagaattgaagaatggcttggcgcaccctgcaagttcatggcagagtctcctattgctgggagtttatctgggaatcctgtgaatcgtgactatatcagacagagacaaggtgcacttgcagggatggagccgaaggaatttcaagccctcaggcaacattcaaaggatgctgggtgtacactagttgaacatattgagtcaccatcgtcaatatgggtatttgctggggcccctgataggtgtccaccaacatgcttgtttgttggagggatggctgagttaggcgccttcttttctatacttcaggatatgaggaacacaatcatggcttcaaaaactgtgggcacagctgatgaaaagcttagaaagaaatcatcattctatcaatcatacctcagacgcacacaatcaatgggaatacaactggaccagaggataattgttatgtttatggttgcctggggaaaggaggcagtggacaactttcatctcggtgatgacatggatccagagcttcgtagcctagctcagatcctgattgaccagaaagtgaaggaaatctcgaaccaggaacctatgaaatta
